# Natural Variation of Lignocellulosic Components in *Miscanthus* Biomass in China

**DOI:** 10.3389/fchem.2020.595143

**Published:** 2020-11-05

**Authors:** Pingping Xu, Senan Cheng, Yanbin Han, Dongbo Zhao, Hongfei Li, Yancui Wang, Guobin Zhang, Cuixia Chen

**Affiliations:** ^1^State Key Laboratory of Crop Biology, Shandong Agricultural University, Taian, China; ^2^College of Agronomy, Shandong Agricultural University, Taian, China

**Keywords:** *Miscanthus*, HPLC, cellulose, hemicellulose, lignin

## Abstract

Lignocellulose content is an important factor affecting the conversion efficiency of biomass energy plants. In this study, 179 *Miscanthus* accessions in China were used to determine the content of lignocellulose components in stems via acid hydrolysis and high-performance liquid chromatography. Results showed that the average lignocellulose content of wild *Miscanthus* germplasm resources was 80.27 ± 6.51%, and the average content of cellulose, hemicellulose, lignin, extracts, and total ash was 38.38 ± 3.52, 24.23 ± 4.21, 17.66 ± 1.56, 14.50 ± 5.60, and 2.53 ± 0.59%, respectively. The average lignocellulose content of *M. sinensis, M. floridulus, M. nudipes, M. sacchariflorus, M. lutarioriparius*, and the hybrids was 77.94 ± 6.06, 75.16 ± 4.98, 75.68 ± 3.02, 83.71 ± 4.78, 81.50 ± 5.23, and 74.72 ± 7.13%, respectively. In all the tested materials, the highest cellulose content was 48.52%, and the lowest was 29.79%. Hemicellulose had the maximum content of 34.23% and a minimum content of 15.71%. The highest lignin content was 23.75%, and the lowest was 13.01%. The lignocellulosic components of different ploidy materials were compared. The content of lignocellulosic components of diploid *M. sacchariflorus* was higher than that of tetraploid *M. sacchariflorus*, and the content of lignocellulosic components of diploid *M. lutarioriparius* was lower than that of tetraploid *M. lutarioriparius*. Analysis of the relationship between the changes in lignocellulosic components and geographical locations of *Miscanthus* showed that the holocellulose and hemicellulose content was significantly positive correlated with the latitude of the original growth location. Results indicated that the lignocellulosic components of *Miscanthus* resources in China are rich in genetic diversity.

## Introduction

*Miscanthus* is a tall perennial herbaceous plant. It belongs to the subtribe *Saccharinae*, tribe *Andropogoneae*, subfamily *Panicoideae*, and family Poaceae. It originated in East Asia and Southeast Asia and is now widely distributed in China, Japan, and Pacific Islands (Hodkinson et al., 2002; Hastings et al., 2009; Jensen et al., 2011) China is an important origin and distribution center of *Miscanthus* (Clifton-Brown et al., 2001; Clifton et al., 2015; Li et al., 2019), with extensive wild germplasm resources and abundant genetic diversity (Hodkinson et al., 2002; Anzoua et al., 2011; Ge et al., 2019). Seven species of *Miscanthus* are found in China, namely, *M. sinensis, M. floridulus, M. sacchariflorus, M. lutarioriparius, M. paniculatus, M. nepalensis*, and *M. nudipes*. The widely distributed species are *M. sinensis, M. floridulus, M. sacchariflorus*, and *M. lutarioriparius*. *Miscanthus* has 19 chromosomes, with diploidy, triploidy, and tetraploidy occurring in nature. *M. sacchariflorus* and *M. lutarioriparius* have both diploid and tetraploid resources (Ge et al., 2017), and natural hybrids exist in nature (Lewandowski et al., 2003; Cichorz et al., 2015).

*Miscanthus* is a lignocellulosic crop with highly efficient C4 photosynthesis, high biomass production, strong stress resistance, and wide adaptability. *Miscanthus* has high water and fertilizer efficiency, excellent cellulose quality, extensive cultivation, eco-friendly environment, and low production costs (Beale and Long, 1995; Clifton-Brown et al., 2001; Lewandowski and Schmidt, 2005; Clifton et al., 2015). The biological yield of *Miscanthus* (3 × 104 kg/ha) is about three times higher than that of switchgrass (Heaton et al., 2008). Compared with other grasses, the lignocellulose of *Miscanthus* is closer to that of wooden materials (Villaverde et al., 2010; Lygin et al., 2011), where the contents of cellulose, hemicellulose, and lignin are ~30–50, 10–40, and 5–30%, respectively (McKendry, 2002; Yang et al., 2007; Kleinert and Barth, 2008). *Miscanthus* is a grassy lignocellulosic material used for converting heat, electricity, and liquid fuels (Cherubini, 2010), as well as for producing aromatic products (Pauly and Keegstra, 2008; Luo et al., 2016; Upton and Kasko, 2016). *Miscanthus* has a higher energy ratio than natural gas and coal, thus it has lower greenhouse gas emissions (Moukamnerd et al., 2010; McCalmont et al., 2017). Compared with high starch or high sugar crops such as sweet sorghum, *Miscanthus* has low moisture and sugar content during harvesting, making it more convenient to store and transport. In addition, unlike cereal crops, harvesting *Miscanthus* as a biofuel does not directly increase the price of cereals (Ziolkowska, 2014). These characteristics make it stand out among many energy crops and have made it one of the most promising non-grain energy plants. Hence, research on *Miscanthus* has sparked wide interest (Feltus and Vandenbrink, 2012; Cao et al., 2019).

With the increase in energy demand, the conversion of lignocellulosic biomass to fuels, such as ethanol, has been the focus of research in many countries. Plants can convert light energy into monosaccharides through photosynthesis and then use CO_2_ to fix monosaccharides into high-energy polymers and generate composite cell walls composed of cellulose, hemicellulose, and lignin (Rubin, 2008). The important factors that make saccharifying lignocellulose raw materials difficult are the degree of polymerization and lignification and cellulose crystallinity (Abramson et al., 2010). Therefore, the main obstacle hindering the accurate determination of lignocellulose content is how to effectively decompose cell walls into fermentable sugars. Five methods are commonly used for determining lignocellulose, including washing cellulose analysis methods, which can measure neutral detergent fiber, acid detergent fiber, and acid detergent lignin. The Klason method is a classic technique for determining lignocellulose, but it overestimates the true lignin value of raw materials. The application of this method is limited because it cannot determine soluble fiber and remove farinaceous substance (Hatfield et al., 1994; Wang et al., 2020). New and state-of-the art technologies, such as near-infrared spectroscopy and nuclear magnetic resonance, have been widely used in determining lignocellulose. Near-infrared spectroscopy can determine the concentration of various plant components, such as fat, grease, protein, and total fiber. However, the detection result of this method is not sufficiently accurate because the spectral measurement value has no direct relation to lignin concentration, and the influence of comparison parameter on the measurement value is high (Li et al., 2015; Ramirez et al., 2015; Hayes et al., 2017; Jin et al., 2017; Elle et al., 2019). Nuclear magnetic resonance spectroscopy is an analytical technique for detecting the composition and structural characteristics of lignin. Considering its inability to obtain good and clear spectra from complex plant samples, this method is currently not widely used (Capanema et al., 2004; Balakshin et al., 2011). The National Renewable Energy Laboratory (NREL) of the United States proposed the NREL method (Sluiter et al., 2008, 2012). Samples are hydrolyzed with sulfuric acid after extracting the extract of the sample to be tested. Glucose content is measured by high-performance liquid chromatography (HPLC). Cellulose content is quantified using the substitution ratio of glucose and cellulose. Lignin content is determined using the differential weight of the residue after hydrolysis of the sample. This method is not only experimentally operable but also provides accurate detection results.

Major breakthroughs in terms of unit biomass production and optimization of biomass conversion efficiency are needed to make the products of second-generation lignocellulosic energy crops economically competitive (Sims et al., 2010; Feltus and Vandenbrink, 2012). The biomass composition of energy plants affects the conversion efficiency. In addition, using *Miscanthus* as a feedstock for bioenergy requires that the biomass composition is adapted to various bioenergy conversion processes (Arnoult and Brancourt-Hulmel, 2015). The development of breeding programs also requires a clear understanding of the content of biomass composition. In order to investigated the biomass composition of *Miscanthus*, the components of lignocellulose in different ecological types of wild resources were determined by using the NREL method. Our research shows that the content of *Miscanthus* lignocellulose is affected by both genetic factors and environmental factors. The results are of great importance for the development and utilization of *Miscanthus* resources in China, genetic breeding of superior energy plants, and the conversion and utilization of biomass energy.

## Materials and Methods

### Materials

From 2011 to 2012, 156 wild *Miscanthus* germplasms in different ecological environments were collected from 23 provinces in China. These germplasms included 5 wild *Miscanthus* species, such as *M. sinensis M. floridulus, M. sacchariflorus, M. lutarioriparius*, and *M. nudipes*, and 23 hybrids. *M. sacchariflorus* and *M. lutarioriparius* have diploid and tetraploid plants in the wild, whereas the other species are only diploid. These materials were planted at the *Miscanthus* germplasm resource nursery (36°09′ N, 117°10′ E) of the Agricultural Experiment Station of Shandong Agricultural University. Each germplasm resource material was subjected to vegetative propagation by subterraneous stem with a planting density of 2 × 2 m. The stems of *Miscanthus* were harvested in March 2013 for the determination of lignocellulosic components.

### Methods

The experimental method followed the NREL method for determining lignocellulose (Thygesen et al., 2005; Sluiter et al., 2012; Kuchelmeister and Bauer, 2015). This method was modified and improved.

#### Sample Pretreatment

Stems were dried to constant weight, crushed, and passed through a 40-mesh sieve. The ground sample (m1 = 0.6 ± 0.010 g) was weighed, reflowed in a Soxhlet extractor (a traditional glass apparatus, Shandong, Hualu) containing water for 8 h, and then dried in a drying oven at 40°C. Then, the sample was refluxed in a Soxhlet extractor containing absolute ethyl alcohol for 16 h and dried in a drying (DHG-9140A, Shanghai) oven at 40°C. After extraction, the remaining solid material (m2) was mainly lignocellulose; the part lost during the process was the extract, and its content was calculated using the equation:

(1) $$\begin{array}{r} \%E=\frac{m1-m2}{m1}\times 100 \end{array}$$

#### Acidolysis of Samples

The extracted sample (m0 = 0.3000 g) was weighed and placed in a pressure-resistant tube (89063-334, VWR). Exactly 3.00 mL 72% H_2_SO_4_ was added, and the mixture was thoroughly stirred and mixed. Then, the sample was placed in a water bath (2321, Fisher Scientific) at 30°C for 60 min. Thereafter, 84.00 mL ddH_2_O was added, and the sample was sterilized in an autoclave (GI80TR, ZEALWAY) (121°C, 1 h).

After acidification of the sample, cellulose was degraded to glucose, whereas hemicellulose was degraded to xylan, arabinose, galactose, and mannose. Lignin was divided into acid-insoluble lignin (AIL) and acid-soluble lignin (ASL). The residue was used for the determination of AIL, whereas the filtrate was used for the determination of ASL and monosaccharides.

#### Determination of Lignin Content

Use a filter crucible (89038-050, VWR) with 15 μm Pore Diameter to filter the hydrolyzed sample to collect the filtrate and residue. AIL was determined via the ashing method. The residue was dried to a constant weight (m3) and then placed in a box-type electrical resistance furnace (SX2-G/T, Shanghai Yuejin). The sample was turned to ash at 575 ± 25°C for 10 h, cooled to room temperature, and weighed (m4). The percent AIL content was determined using the equation :

(2) $$\begin{array}{r} \%AIL=\frac{m3-m4}{m0}\times \left(1-E\right)\times 10 \end{array}$$

ASL was determined using an UV-Vis spectrophotometer (Nanodrop2000c, Thermo). The absorbance of the filtrate was determined at λ = 205 nm. The percent ASL content was calculated using the equation:

(3) $$\begin{array}{r} \%ASL=\frac{\varepsilon \times D\times V}{K\times m0\times 1000}\times \left(1-E\right)\times 100 \end{array}$$

Where ε represents the absorption value, *D* represents dilution factor, *V* is the total liquid volume (87 mL), and *K* = 110 represents the absorption coefficient of acid-soluble lignin (Hayes, 2012).

Lignin content was calculated using the equation:

(4) $$\begin{array}{r} \%Lignin\;=\;\%AIL\;+\;\%ASL \end{array}$$

#### Determination of Cellulose and Hemicellulose

Monosaccharide content was determined via HPLC [Chromatographic conditions: chromatographic column (Biorad Aminex HPX-87P), Deashing packed column, Detector (evaporative light scattering detector), Injection volume (35 μL), mobile phase (Ultrapure water), flow velocity (0.6 mL/min), Nitrogen pressure (30 psi); drift tube (heating mode, 80 ± 25°C), Sprayer (60%); running time (20min)]. Exactly 4 mL of the filtrate was obtained, and the pH was adjusted to 5–6 with CaCO_3_. The supernatant was collected by centrifugation and filtered through a 0.22 μm filter membrane. Then, HPLC was used to determine the content of monosaccharides. Both monosaccharides and calcium carbonate are pure reagents (Sigma) for chromatographic analysis.

The cellulose and hemicellulose contents were calculated from the monosaccharide content as follows:

(5) $$\begin{array}{r} \%Cellulose\;=\;\%Glu\;\times \;Ac \end{array}$$

(6) $$\begin{array}{l} \%Hemicellulose\;=\;\%Xylose\;\left(Xyl\right)\times \;Ac\;+\;\%Arabinose\;\left(Ara\right)\\ \;\times \;Ac\;+\;\%Galactose\;\left(Gal\right)\times \;Ac\\ \;+\;\%Mannose\;\left(Man\right)\times \;Ac \end{array}$$

(7) $$\begin{array}{r} \%\;Holocellulose\;=\;\%Cellulose\;+\;\%Hemicellulose \end{array}$$

where Ac is the dehydration correction coefficient. The Ac values of pentose and hexose were 0.88 and 0.90, respectively. %Glu, %Xyl, %Ara, %Gal, and %Man represent the contents of the corresponding monosaccharides obtained by the regression curve method.

#### Determination of Total Ash

Weigh the mass of the empty crucible, record it as m5. Then weigh about 0.5 g of the sample, put it in a filter crucible and weigh it (m6), then put it into a box-type electric furnace, 575 ± 25°C, 24 h, cool it to room temperature in a desiccator and weigh m7.

(8) $$\begin{array}{r} \%H=\frac{m7-m5}{m6-m5}\times 100 \end{array}$$

### Data Processing

The determination results of each *Miscanthus* sample were expressed as the average of three replicates. Data statistics were completed and coefficient of variation was calculated using Excel. Maps, mapdata, and ggplot2 software packages in R (3.6.0) were used to draw the distribution of material sources. SPSS software (statistics 24.0) was used to perform single-factor ANOVA test and obtain the boxplot of component content. Pearson correlation analysis was used to determine the material geographic location and lignocellulosic component content.

## Result

### Original Geographic Distribution of Materials

**Figure 1:**
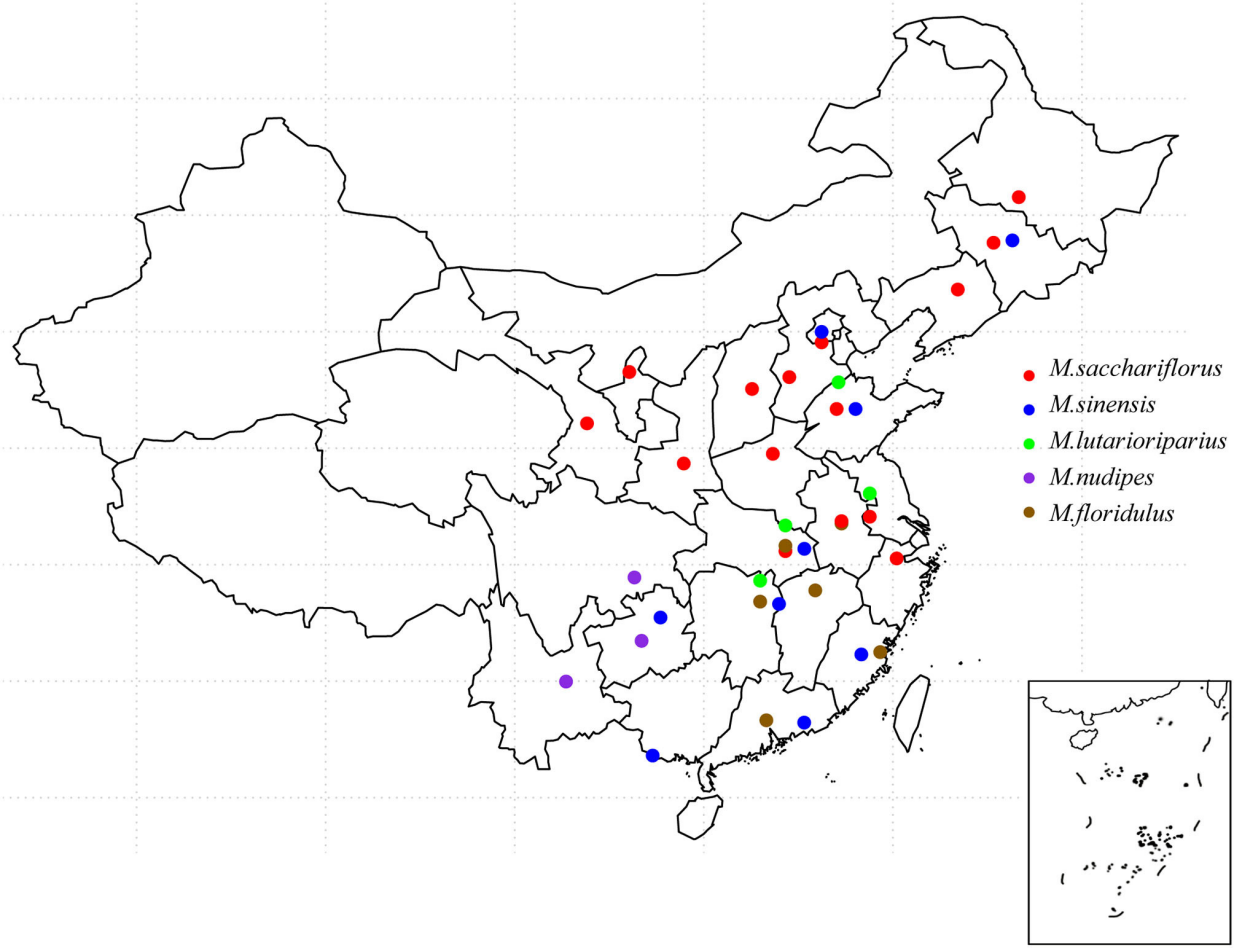
Distribution of *Miscanthus* species in China. The picture on the bottom right shows the South China Sea Islands.

The original location of the *Miscanthus* accessions analyzed in this study included 23 provinces (spanning 21°31′ N, 46°07′ N from south to north and 102°32′ E, 128°91′ E from west to east, with an altitude of 1–1,650 m above sea level). The experimental materials contained 86 *M. sacchariflorus* materials, among which 72 were diploid and 14 were tetraploid. *M. lutarioriparius* had eight accessions of diploid and eight accessions of tetraploid. We found 31 *M. sinensis*, 19 *M. floridulus*, 23 hybrids, 4 *M. nudipes*. Among them, tetraploid *M. sacchariflorus* was mainly distributed in Shandong and Henan Provinces, whereas tetraploid *M. lutarioriparius* was mostly distributed in Hunan, Jiangsu, and Hubei Provinces. It can be seen from the distribution map that the distribution of five species of *Miscanthus* in China has a certain regionality. It is not difficult to see that the distribution range of *M. sinensis* and *M. sacchariflorus* is the widest, while the distribution range of *M. nudipes* is relatively concentrated. In addition, the sources and detailed distribution of all materials are shown in Supplemental Material. These materials cover the main distribution areas of *Miscanthus* species in China (Figure 1).

### Analysis of Lignocellulosic Components of *Miscanthus*

**Figure 2:**
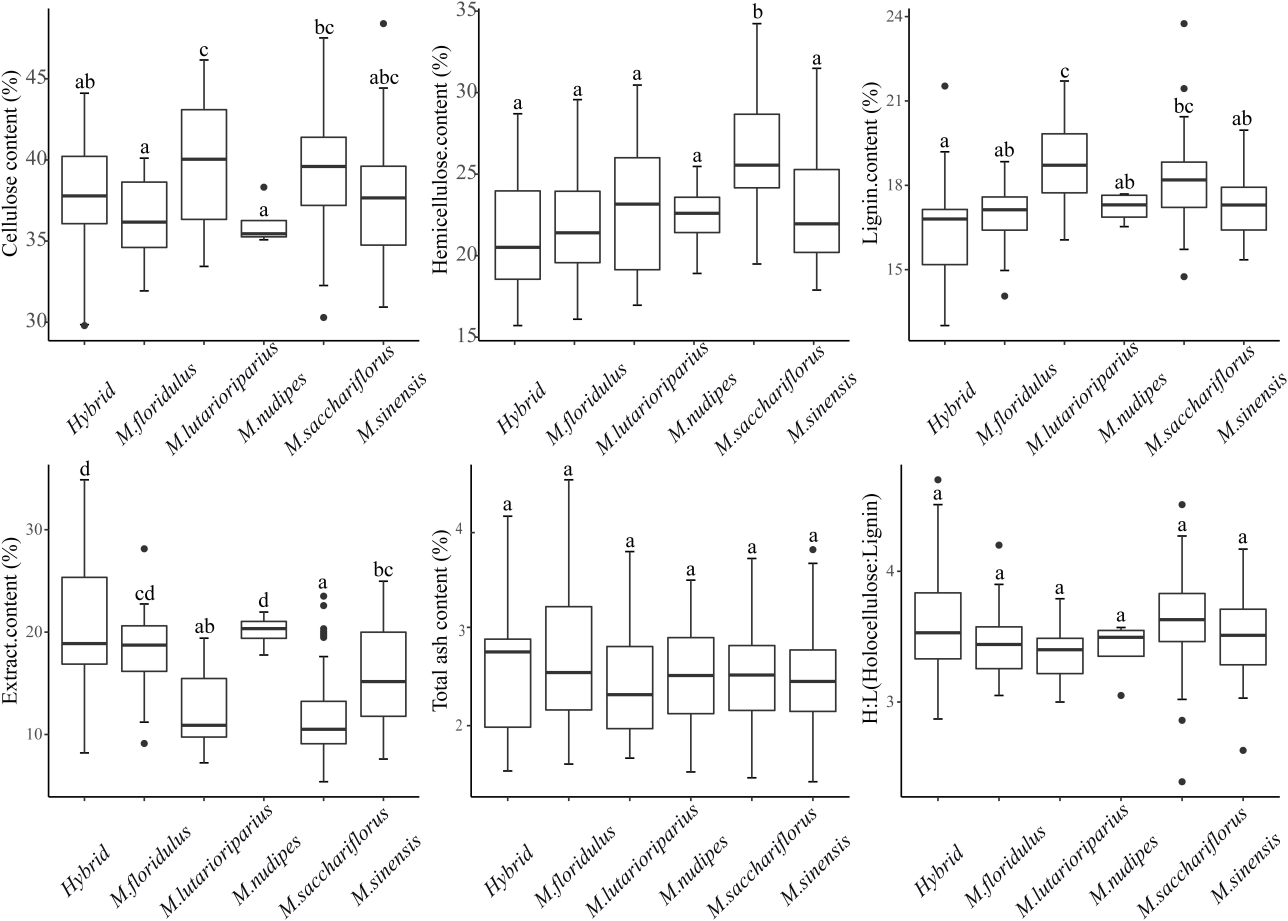
Statistical comparison of stem components in different *Miscanthus* species. Different small letters indicate significant differences at the *P* < 0.05 level. The upper limit of Whisker represents the largest non-outlier value, and the lower limit is the smallest non-outlier value. The upper border line of the box represents the upper quartile, the middle line represents the median, and the lower border line represents the lower quartile. The dots around the box represent outliers.

The results of the determination of lignocellulosic components of 179 *Miscanthus* materials showed that the average content of all lignocelluloses (the sum of cellulose, hemicellulose, and lignin) was 80.27 ± 6.51%, of which the content of cellulose, hemicellulose, lignin, extracts, and total ash was 38.38 ± 3.52, 24.23 ± 4.21, 17.66 ± 1.56, 14.50 ± 5.60, and 2.53 ± 0.59%, respectively. The average lignocellulose content (the sum of cellulose, hemicellulose, and lignin) of *Miscanthus* can be arranged in descending order as follows: *M. sacchariflorus* (83.71 ± 4.78%), *M. lutarioriparius* (81.50 ± 5.23%), *M. sinensis* (77.94 ± 6.06%), *M. nudipes* (75.68 ± 3.02%), *M. floridulus* (75.16 ± 4.98%), and hybrids (74.72 ± 7.13%) (Table 1, Supplementary Table 1). Univariate analysis of variance was used to analyze the difference in content among species (Figure 2), and the difference in lignocellulosic compositions of *Miscanthus* species was analyzed using the coefficient of variation to determine their potential genetic diversity (Table 2).

**Table 1:** Statistics on lignocellulose content of *Miscanthus*.

**Species**	**Lignocellulose**	**Cellulose**	**Hemicellulose**	**Lignin**	**Extracts**	**Total ash**	**H/L**
*M. sinensis*	77.94 ± 6.06%	37.66 ± 3.80%	22.94 ± 3.71%	17.35 ± 1.29%	15.83 ± 5.02%	2.47 ± 0.57%	3.51 ± 0.36
*M. floridulus*	75.16 ± 4.98%	36.28 ± 2.58%	21.95 ± 3.61%	16.94 ± 1.18%	18.41 ± 4.17%	2.74 ± 0.81%	3.45 ± 0.29
*M. nudipes*	75.68 ± 3.02%	36.07 ± 1.51%	22.39 ± 2.70%	17.21 ± 0.55%	21.10 ± 1.77%	2.51 ± 0.81%	3.40 ± 0.24
*M. sacchariflorus*	83.71 ± 4.78%	39.25 ± 3.06%	26.35 ± 3.73%	18.11 ± 1.35%	11.62 ± 3.81%	2.51 ± 0.48%	3.64 ± 0.34
*M. lutarioriparius*	81.50 ± 5.23%	39.96 ± 3.96%	22.85 ± 3.95%	18.69 ± 1.49%	12.43 ± 3.84%	2.43 ± 0.65%	3.37 ± 0.22
Hybrid	74.72 ± 7.13%	37.14 ± 3.98%	21.21 ± 3.53%	16.37 ± 1.95%	20.67 ± 6.43%	2.56 ± 0.72%	3.60 ± 0.46
Average	80.27 ± 6.51%	38.38 ± 3.52%	24.23 ± 4.21%	17.66 ± 1.56%	14.50 ± 5.60%	2.53 ± 0.59%	3.56 ± 0.35

*H/L, Holocellulos/Lignin. Same as below*.

**Table 2:** Coefficient of variation of lignocellulosic components of *Miscanthus*.

**Species**	***M. sinensis***	***M. floridulus***	***M. nudipes***	***M. sacchariflorus***	***M. lutarioriparius***	**Hybrid**
Cellulose	10.09%	7.12%	4.19%	7.80%	9.91%	10.73%
Hemicellulose	16.18%	16.46%	12.07%	14.14%	17.29%	16.65%
Lignin	7.44%	6.98%	3.22%	7.45%	7.98%	11.91%
Extracts	31.72%	22.66%	8.78%	32.81%	30.89%	31.09%
Total ash	23.14%	29.36%	32.33%	19.04%	26.88%	28.09%
H/L	10.36%	8.45%	7.02%	9.29%	6.48%	12.87%

*Coefficient of variation (CV) = (Standard deviation/Mean) × 100%*.

The average cellulose content of *M. sinensis, M. floridulus, M. nudipes, M. sacchariflorus, M. lutarioriparius*, and the hybrids was 37.66 ± 3.80, 36.28 ± 2.58, 36.07 ± 1.51, 39.25 ± 3.06, 39.96 ± 3.96, and 37.14 ± 3.98%, respectively. Among all *Miscanthus* plant materials, M020 (*M. sinensis*, from Fujian Province) had the highest cellulose content of 48.52%, whereas M311 (hybrid, from Hunan Province) had the lowest cellulose content of 23.62% (Table 1, Supplementary Table 1). One-way ANOVA test found that the cellulose content of *M. lutarioriparius* was significantly different from that of *M. floridulus, M. nudipes*, and the hybrids but not significantly different from that of *M. sinensis* and *M. sacchariflorus* (Figure 2). The coefficients of variation were ranked from large to small as follows: hybrids (10.73%), *M. sinensis* (10.09%), *M. lutarioriparius* (9.91%), *M. sacchariflorus* (7.80%), *M. floridulus* (7.12%), and *M. nudipes* (4.19%).

The average hemicellulose content of *M. sinensis, M. floridulus, M. nudipes, M. sacchariflorus, M. lutarioriparius*, and the hybrids was 22.94 ± 3.71, 21.95 ± 3.61, 22.39 ± 2.70, 26.35 ± 3.73, 22.85 ± 3.95, and 21.21 ± 3.53%, respectively. Among all the tested materials of *Miscanthus*, M137 (*M. sacchariflorus*, from Liaoning Province) had the highest hemicellulose content of 34.23%, whereas M010 (hybrid, from Hunan Province) had the lowest hemicellulose content of 15.71% (Table 1, Supplementary Table 1). The hemicellulose content of *M. sacchariflorus* was significantly different from that of *M. sinensis, M. floridulus, M. lutarioriparius, M. nudipes*, and the hybrids based on the results of ANOVA test (Figure 2). The coefficient of variation of hemicellulose can be arranged as follows: *M. lutarioriparius* (17.29%), hybrids (16.65%), *M. floridulus* (16.46%), *M. sinensis* (16.18%)*, M. sacchariflorus* (14.14%), and *M. nudipes* (12.07%).

The average lignin content of *M. sinensis, M. floridulus, M. nudipes, M. sacchariflorus, M. lutarioriparius*, and the hybrids was 17.35 ± 1.29, 16.94 ± 1.18, 17.21 ± 0.55, 18.11 ± 1.35, 18.69 ± 1.49, and 16.37 ± 1.95%, respectively. Of all the determined plant materials, the lignin content of M123 (*M. sacchariflorus*, from Heilongjiang Province) was the highest at 23.75%, whereas the lignin content of M322 (hybrid, from Hunan Province) was the lowest at 13.01% (Table 1, Supplementary Table 1). ANOVA test revealed significant differences between the lignin content of *M. lutarioriparius* and that of *M. sinensis, M. floridulus, M. nudipes*, and the hybrids. The difference between *M. lutarioriparius* and *M. sacchariflorus* was not significant (Figure 2). The coefficient of variation of lignin was 11.91% for the hybrids, 7.98% for *M. lutarioriparius*, 7.45% for *M. sacchariflorus*, 7.44% for *M. sinensis*, 6.98% for *M. floridulus*, and 3.22% for *M. nudipes*.

The average content of the extracts of *M. sinensis, M. floridulus, M. nudipes, M. sacchariflorus, M. lutarioriparius*, and the hybrids was 15.83 ± 5.02, 18.41 ± 4.17, 21.10 ± 1.77, 11.62 ± 3.81, 12.43 ± 3.84, and 20.67 ± 6.43%, respectively. Among all the tested species, M322 (hybrid, from Hunan Province) had the highest extract content of 34.88%, whereas M177 (*M. sacchariflorus*, from Shandong Province) had the lowest extract content of 5.38% (Table 1, Supplementary Table 1). In the ANOVA test, the contents of extracts of *M. nudipes* and hybrids were significantly different from *M. sinensis, M. floridulus, M. sacchariflorus, M. lutarioriparius*, respectively (Figure 2). The coefficient of variation of the extracts had the largest difference, and this parameter cam be arranged in the following order: *M. sacchariflorus* (32.81%), *M. sinensis* (31.72%), hybrids (31.09%), *M. lutarioriparius* (30.89%), *M. floridulus* (22.66%), and *M. nudipes* (8.78%). The total ash content of *M. sinensis, M. floridulus, M. nudipes, M. sacchariflorus, M. lutarioriparius*, and the hybrids was 2.47 ± 0.57, 2.74 ± 0.81, 2.51 ± 0.81, 2.51 ± 0.48, 2.43 ± 0.65, and 2.56 ± 0.72%, respectively. Among all the measured materials, M214 (*M. sinensis*, from Zhejiang Province) had the highest total ash content of 4.5%, whereas M165 (*M. sinensis*, from Guangxi) had the lowest total ash content of 1.43% (Table 1, Supplementary Table 1). ANOVA test showed no significant difference in total ash content between each species (Figure 2). The order of coefficient of variation of total ash content can be arranged as follows: *M. nudipes* (32.33%), *M. floridulus* (29.36%), hybrid (28.09%), *M. lutarioriparius* (26.88%), *M. sinensis* (23.14%), and *M. sacchariflorus* (19.04%).

The holocellulose-to-lignin (H/L) ratio of *M. sinensis, M. floridulus, M. nudipes, M. sacchariflorus, M. lutarioriparius*, and the hybrids was 3.51 ± 0.36, 3.45 ± 0.29, 3.40 ± 0.24, 3.64 ± 0.34, 3.37 ± 0.22, and 3.60 ± 0.46, respectively. M171 (hybrid, from Hunan Province) had the highest value at 4.70, whereas M123 (*M. sacchariflorus*, from Heilongjiang Province) had the lowest value at 2.39 (Table 1, Supplementary Table). ANOVA test showed that the H/L ratio did not differ significantly between species (Figure 2). The coefficient of variation of H/L ratios can be arranged as follows: hybrids (12.87%) > *M. sinensis* (10.36%) > *M. sacchariflorus* (9.29%) > *M. floridulus* (8.45%) > *M. nudipes* (7.02%) > *M. lutarioriparius* (6.48%).

### Comparative Analysis Between Different Ploidies of *M. sacchariflorus* and *M. lutarioriparius*

A total of 86 parts of *M. sacchariflorus* (including 72 diploids and 14 tetraploids) and 18 parts of *M. lutarioriparius* (eight parts of diploids and eight parts of tetraploids) were collected for this test. We conducted a comparative analysis of the materials with different ploidies (Table 3). We found that diploid *M. sacchariflorus* had higher lignocellulose content than tetraploid *M. sacchariflorus* (4X), whereas diploid (2X) *M. lutarioriparius* had less lignocellulose content than tetraploid (4X) *M. lutarioriparius*. The content of cellulose, hemicellulose, and lignin of diploid *M. sacchariflorus* ranged from 32.60 to 47.52, 19.49 to 34.23, and 14.75 to 23.75%, respectively, whereas that of tetraploid *M. sacchariflorus* ranged from 30.29 to 42.63, 20.23 to 33.55, and 17.14 to 20.28%, respectively. The content of cellulose, hemicellulose, and lignin ranged from 35.46 to 46.16, 16.96 to 26.73, and 16.06 to 21.71%, respectively, in diploid *M. lutarioriparius*, whereas it ranged from 33.44 to 44.87, 18.74 to 30.46, and 17.78 to 20.31%, respectively, in triploid *M. lutarioriparius*.

**Table 3:** Statistical results of lignocellulose fractions of different ploidies of *M. sacchariflorus* and *M. lutarioriparius*.

**Component**	**Species**	**Ploid**	**Average value (%)**	**Coefficient of variation (%)**	**Minimum (%)**	**Maximum (%)**
Lignocellulose	*M. sacchariflorus*	2	84.05 ± 4.89	5.82	72.47	91.79
		4	81.97 ± 3.87	4.73	72.41	85.90
	*M. lutarioriparius*	2	78.65 ± 5.64	6.66	69.46	86.82
		4	84.35 ± 3.56^*^	4.23	77.41	89.99
Cellulose	*M. sacchariflorus*	2	39.58 ± 2.73	6.89	32.60	47.52
		4	37.53 ± 4.10	10.93	30.29	42.63
	*M. lutarioriparius*	2	39.60 ± 4.03	10.19	35.46	46.16
		4	40.33 ± 4.12	10.23	33.44	44.87
Hemicellulose	*M. sacchariflorus*	2	26.46 ± 3.58	13.52	19.49	34.23
		4	25.83 ± 4.54	17.56	20.23	33.55
	*M. lutarioriparius*	2	20.74 ± 3.41	16.44	16.96	26.73
		4	24.96 ± 3.42^*^	13.69	18.74	30.46
Lignin	*M. sacchariflorus*	2	18.01 ± 1.40	7.78	14.75	23.75
		4	18.6 ± 0.92	4.97	17.14	20.28
	*M. lutarioriparius*	2	18.31 ± 1.87	10.21	16.06	21.71
		4	19.07 ± 0.97	5.10	17.78	20.31
Holocellulose	*M. sacchariflorus*	2	66.04 ± 4.52	6.84	55.92	73.98
		4	63.37 ± 3.73^*^	5.89	53.64	67.56
	*M. lutarioriparius*	2	60.34 ± 3.73	6.18	53.40	65.12
		4	65.28 ± 3.17^*^	4.86	59.63	69.68
Extracts	*M. sacchariflorus*	2	11.42 ± 4.03	35.25	5.38	23.51
		4	12.64 ± 2.27	17.99	9.28	17.35
	*M. lutarioriparius*	2	14.29 ± 4.05	28.33	8.10	19.41
		4	10.57 ± 2.71^*^	25.62	7.23	16.46
Total ash	*M. sacchariflorus*	2	2.58 ± 0.47	18.04	1.51	3.70
		4	2.14 ± 0.37^**^	17.09	1.47	2.83
	*M. lutarioriparius*	2	2.22 ± 0.37	16.73	1.67	2.67
		4	2.65 ± 0.82	30.97	1.73	3.77
H/L	*M. sacchariflorus*	2	3.68 ± 0.34	9.14	2.39	4.51
		4	3.41 ± 0.25^*^	7.46	2.86	3.73
	*M. lutarioriparius*	2	3.31 ± 0.22	6.76	3.00	3.57
		4	3.43 ± 0.21	6.14	3.15	3.79

* *Significant difference at P < 0.05*,

** *Significant difference at P < 0.01*.

A comparison of the content of lignocellulosic components revealed significant differences in total cellulose content and H/L index and very significant differences in total ash content between 2X *M. sacchariflorus* and 4X *M. sacchariflorus*. The content of hemicellulose, holocellulose, lignocellulose, and extracts of 2X *M. lutarioriparius* was significantly different from that of 4X *M. lutarioriparius*. The coefficient of variation of cellulose and hemicellulose content of 2X *M. sacchariflorus* was less than that of 4X *M. sacchariflorus*, whereas that of lignin content of 2X *M. sacchariflorus* was greater than that of 4X *M. sacchariflorus*. The coefficient of variation of cellulose and lignin content of 2X *M. lutarioriparius* was less than that of 4X *M. lutarioriparius*, whereas the coefficient of variation of hemicellulose content of 2X *M. lutarioriparius* was greater than that of 4X *M. lutarioriparius*. Owing to ploidy changes, the cellulose and hemicellulose content of 4X *M. sacchariflorus* tended to diversify, whereas and its lignin content was more stable. The cellulose and lignin content of 4X *M. lutarioriparius* tended to diversify, whereas its hemicellulose content remained stable.

### Comparative Analysis of *Miscanthus* Materials From Different Ecological Regions

**Figure 3:**
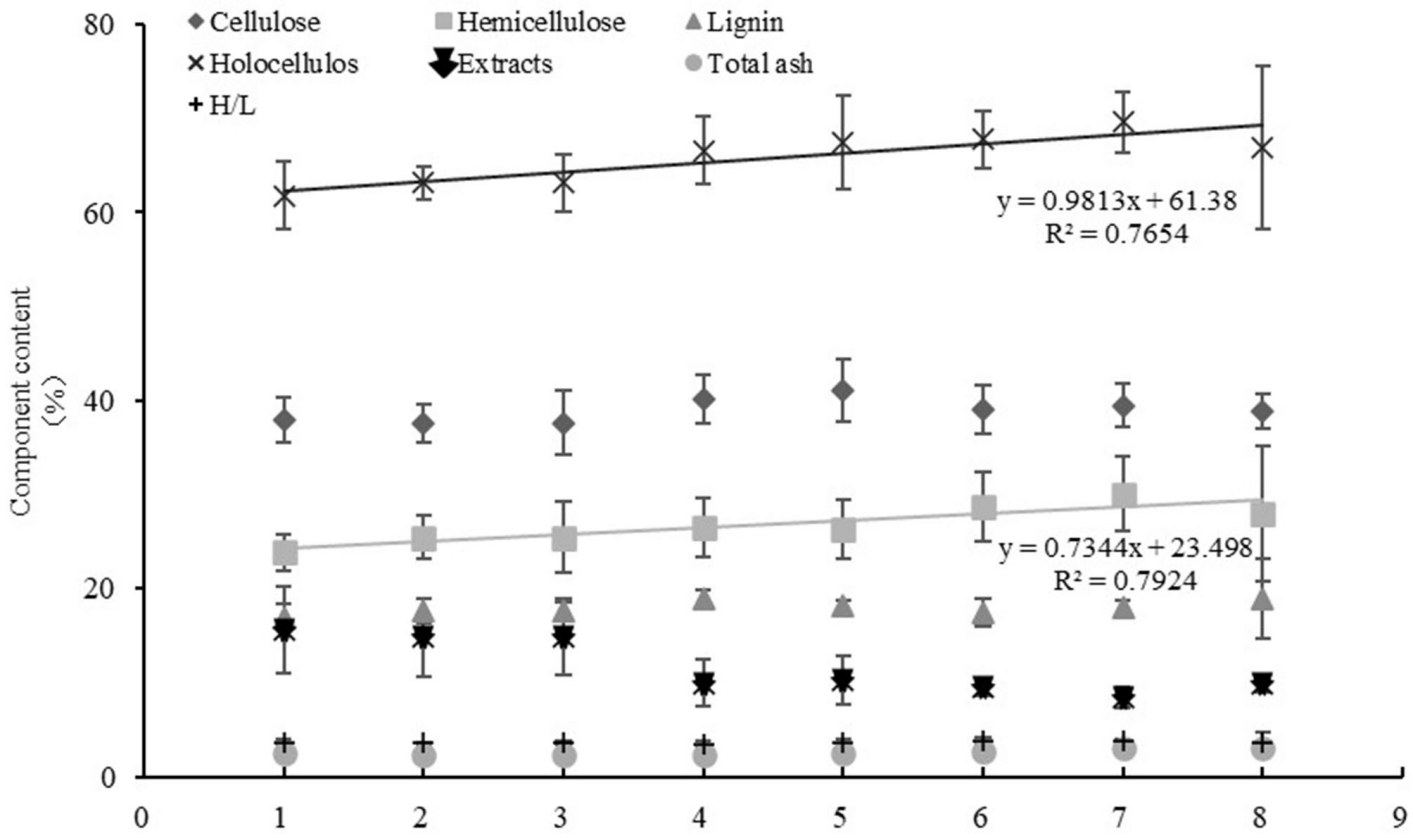
Linear analysis of latitude and lignocellulosic components. 1: 30–32 N°, 2: 32–34 N°, 3: 34–36 N°, 4: 36–38 N°, 5: 38–40 N°, 6: 40–42 N°, 7: 42–44 N°, 8: 44–46 N°.

The correlation between the differences in geographical factors (longitude, latitude, and altitude) and lignocellulosic components of *M. sacchariflorus* were analyzed. Results showed that the hemicellulose and holocellulose content increased with the increase in latitude, and a very significant positive correlation with latitude was observed. The correlation coefficients were 0.409 and 0.441 for hemicellulose and holocellulose, respectively. A significant positive correlation was found between total ash content and longitude and latitude, with correlation coefficients of 0.342 and 0.361, respectively. The extract content had a significant negative correlation with longitude and latitude, with coefficients of −0.346 and −0.523, respectively. In addition, no correlation was found between each lignocellulosic component of *Miscanthus* and altitude (Table 4). Further linear analysis was performed. Results showed that the difference in latitude would cause changes in hemicellulose and holocellulose content (the sum of hemicellulose and cellulose). The values of *R*^2^ were 0.7924 and 0.7654, respectively, indicating that the linear fitting equation was credible, and the high credibility reflects the correlation between total cellulose and hemicellulose content and latitude (Figure 3). This result was consistent with that of Pearson correlation analysis.

**Table 4:** Correlation analysis between lignocellulose content and geographical distribution of *M. sacchariflorus*.

***M. sacchariflorus***	**Latitude**		**Longitude**		**Altitude**	
***N* = 85**	**Pearson**	**Significance**	**Pearson**	**Significance**	**Pearson**	**Significance**
	**correlation**		**correlation**		**correlation**	
Cellulose	0.147	0.178	−0.060	0.586	0.171	0.119
Hemicellulose	0.409^**^	0.000	0.242^**^	0.026	−0.007	0.948
Lignin	0.162	0.138	0.113	0.304	−0.080	0.466
Holocellulose	0.441^**^	0.000	0.160	0.145	0.111	0.311
Extracts	−0.523^**^	0.000	−0.346^**^	0.001	0.095	0.389
Total ash	0.361^**^	0.001	0.342^**^	0.001	−0.094	0.393
H/L	0.239^*^	0.028	0.073	0.507	0.131	0.234

* *Significant difference at P < 0.05*,

** *Significant difference at P < 0.01*.

## Discussion

### Effects of Geographical Factors on Lignocellulose Content

China is an important place of origin and distribution center of *Miscanthus*, with extensive wild germplasm resources and abundant genetic diversity. Four species of *Miscanthus* are found in China, namely, *M. sinensis, M. sacchariflorus, M. lutarioriparius*, and *M. floridulus*. *M. sinensis* and *M. sacchariflorus* are widely distributed in northern and southern China. *M. lutarioriparius* and *M. floridulus* are mainly located south of the Yangtze River, where the climate is relatively warm. *M. lutarioriparius* is a unique variant in China and the primary raw material for papermaking in the country. *M. sacchariflorus* and *M. lutarioriparius* have two types of diploids and tetraploids in China. The tetraploid *M. sacchariflorus* is mainly distributed in Shandong and Henan Provinces, whereas the tetraploid *M. lutarioriparius* is mainly distributed in Hunan, Jiangsu, and Hubei Provinces. Few other species of *Miscanthus*, such as *M. nudipes*, are found in the arid mountainous areas at high altitudes in southwest China. We found that related geographical factors, such as latitude, had an important selection effect on *Miscanthus* species, and the holocellulose and hemicellulose content increased with latitude. This result was consistent with that obtained by Zhao et al. (2014). Youngmi Kim also found similar patterns on the lignocellulose content of switchgrass (Kim et al., 2011). Low temperature increases the content of soluble sugar in plants to protect the stability of cell membranes (Jan et al., 2009; Pompeiano et al., 2015). Papini-Terzi found a correlation between some genes associated with cell wall metabolism and sugar content in plants. Plants under cold weather conditions increase their soluble sugar content, thereby indirectly inducing the synthesis of related lignocelluloses (Papini-Terzi et al., 2009; Vicentini et al., 2009; Waclawovsky et al., 2010). The holocellulose content of *Miscanthus* in high latitudes is relatively high. A statistical field phenotypic survey revealed significant differences in the flowering and maturity stages of *Miscanthus* plants transplanted to the Shandong experimental base due to the influence of photoperiod (Imaizumi and Kay, 2006). Light time increases with the increase in latitude, resulting in short flowering time. Analysis of *M. sacchariflorus* showed that in the Yangtze River Basin and south of the region, the flowering period is mainly concentrated in mid-to-late September and early October, but the flowering period in Shandong and Beijing mainly occurs in mid-to-late August and early September. Blooming in Heilongjiang, Jilin, and Liaoning mostly occurs in mid-to-late June. Hence, geographical location has an obvious selective effect on the genetic variation in *Miscanthus*, and this variation is closely related to phenological conditions corresponding to geographical location, such as photoperiod, accumulated temperature, and rainfall. Considering the vastness of China's geographical locations, the species of *Miscanthus* in China have rich diversity.

### Comparative Analysis of the Determination Results of Lignocellulosic Components of *Miscanthus*

Jung compared the lignocellulose content of *Miscanthus*, switchgrass, sorghum, and reeds and found that the lignin content of *Miscanthus* was significantly lower than that of reeds. Therefore, *Miscanthus* is a more suitable energy plant than reeds. Moreover, *M. sacchariflorus* contains 14.12% lignin and 64.23% holocellulose (Heaton, 2008). Kim determined the lignocellulose content of 12 species of *Miscanthus*, including *M. sinensis, M. sacchariflorus*, and *Miscanthus* × *giganteus*, with a cellulose content of 36.1–44.9%, hemicellulose content of 17.1–30.5%, and lignin content of 13.8–31.1% (Kim et al., 2012). The cellulose, hemicellulose, and lignin content of all *Miscanthus* plants measured in this test ranged from 29.79 to 48.52, 15.71 to 34.23, and 13.01 to 23.75%, respectively. The measured lignin content was slightly lower than that obtained by Kim probably due to differences in measurement methods. The rest of the results are consistent with those of previous studies. The value range of the content of lignocellulosic components of *Miscanthus* plants is quite different, a result that also confirms the richness of *Miscanthus* germplasm resources in China.

The content of lignocellulosic components of *Miscanthus* plants has obvious differences within species. For example, the cellulose, hemicellulose, and lignin content of *M. sinensis* ranged from 30.93 to 48.40, 17.89 to 31.49, and 15.35 to 19.96%, respectively, whereas that of *M. sacchariflorus* ranged from 30.29 to 47.52, 19.49 to 34.23, and 14.75 to 23.75%, respectively. The maximum cellulose, hemicellulose, and lignin content was 1.5 times or higher than it, indicating that *Miscanthus* plants have abundant diversity within each species. Obvious differences were observed among various species of *Miscanthus*. For example, the content of hemicellulose and other components of *M. sacchariflorus* was considerably different from that of *M. sinensis* and *M. floridulus* according to single-factor ANOVA analysis. Significant differences were also observed in cellulose and lignin content among species. However, the contents of major lignocellulosic components, such as cellulose, hemicellulose, and lignin, between *M. sinensis* and *M. floridulus* and between *M. sacchariflorus* and *M. lutarioriparius* were slightly different. This result arose because the evolutionary relationship between *M. sinensis* and *M. floridulus* is close, as well as the genetic evolution between *M. sacchariflorus* and *M. lutarioriparius*. Ge described this evolutionary relationship in detail (Ge et al., 2017).

The coefficient of variation was calculated to determine the difference and potential genetic diversity of the various lignocellulosic components of *Miscanthus*. Generally, the coefficient of variation of each species of *Miscanthus* was high, indicating that its lignocellulosic components in China have rich diversity. The coefficient of variation of the content of each component of the hybrids was high in all species, proving that selecting varieties with high cellulose and hemicellulose contents and low lignin content from hybrids is easier than from wild types. The coefficient of variation of each component (except total ash) of *M. nudipes* was relatively low among all species, and this observation may explain the relatively concentrated geographical distribution of this species.

### Significance and Application of the Determination of Lignocellulose Content of *Miscanthus*

In a 3-year field trial in Illinois, USA, Jung found that *Miscanthus* × *giganteus* has a biotransformation efficiency about 2.7 times higher than that of corn (Jung et al., 2015). Therefore, *Miscanthus* is widely studied as a second-generation biomass energy source. Lignocellulosic biomass is mainly a complex structure composed of cellulose, hemicellulose, lignin, and some extractable components. The amount, proportion, and type of each ingredient largely depend on the type of raw material (Pauly and Keegstra, 2008; Zhang et al., 2012; Yu et al., 2018). Cellulose and hemicellulose belong to polysaccharides. Cellulose and hemicellulose are used for the conversion of biomass energy, and their content determines the efficiency of fuel conversion (Bosch and Hazen, 2013). Lignin is an amorphous high-molecular organic polymer with a three-dimensional network structure composed of carbon–oxygen and carbon–carbon bonds. It cross-links cellulose and hemicellulose to provide good support for the stem. The complex chemical structure of lignin hinders the degradation of cellulose and hemicellulose and makes the conversion and use of biomass energy difficult (Boudet et al., 2003; Sticklen, 2006, 2008; Chang, 2007; Chen and Dixon, 2007; Li et al., 2008). Therefore, choosing varieties with high cellulose and hemicellulose content and low lignin content is beneficial to improve energy conversion efficiency. In view of this analysis, we proposed the H/L index, which can reflect the difficulty in converting cellulose energy plants into energy substances to a certain extent. *Miscanthus* with a high H/L value is suitable for conversion to alcohols by fermentation. The H/L value of *Miscanthus* did not significantly differ among species, but obvious differences were observed among varieties. The H/L index also has an important reference value in breed selection.

*M. giganteus* is the most studied species in terms of production applications. *M. giganteus* has high cellulose content and strong adaptability. Hence, it is widely cultivated in European countries. We obtained 23 hybrids (*M. sinensis* × *M. sacchaflorus, M. floridulus* × *M. sacchariflorus*, and *M. floridulus* × *M. lutarioriparius*) through artificial crosses. The yield of hybrids has obvious advantages compared with other *Miscanthus* species. Moreover, the lignin content of the hybrids was higher than that of *M. sinensis* and *M. floridulus*. Therefore, selecting varieties with high total cellulose content and low lignin content from hybrids is easier than from wild types. Hence, artificial hybrid breeding is an effective way of selecting excellent energy plants. According to the determination results of lignocellulose combined with the growth adaptability characteristics of *Miscanthus*, and by making full use of the abundant resources of *Miscanthus* in China, selective artificial breeding was performed to select energy plants with optimized content of stem components and broad growth adaptability.

## Conclusion

From the overall results, *Miscanthus* is a good bioenergy plant with high lignocellulose content. At present, the biological production of various platform chemicals has been realized, such as ethanol, butanol, lactic acid, levulinic acid, sorbitol, glycerol, 1,3-propanediol, itaconic acid, succinic acid, and 2,5-FDCA. Therefore, suitable accessions of *Miscanthus* can be selected based on their biomass composition and different transformation processes. Further analyses found that *Miscanthus* there is obvious differences among intra- and interspecies of *Miscanthus*, and the content of lignocellulosic components has a wide range of values, in consistent with rich genetic diversity. More interestingly, our research shows that the content of *Miscanthus* lignocellulose is not only affected by genetic factors (such as ploidy), but also by environmental factors, such as Miscanthus in high latitudes with higher hemicellulose content. Overall, this research laid a solid foundation for the efficient development of *Miscanthus* biomass conversion, genetic breeding, and utilization in the future.

## Data Availability Statement

The original contributions presented in the study are included in the article/Supplementary Material, further inquiries can be directed to the corresponding author/s.

## Author Contributions

PX and SC analysis of experimental data and article writing. HL and DZ collection of experimental materials. YH and YW designed the experimental method. CC and GZ guidance of experimental ideas. All authors contributed to the article and approved the submitted version.

## Conflict of Interest

The authors declare that the research was conducted in the absence of any commercial or financial relationships that could be construed as a potential conflict of interest.
