# Supplementary material for: Natural Variation of Lignocellulosic Components in Miscanthus Biomass in China
Source: Front Chem. 2020 Nov 5;8:595143. doi: 10.3389/fchem.2020.595143 (PMC7674668; doi:10.3389/fchem.2020.595143)
Supplement: Supplementary file 1 [file Table_1.DOCX]

**Table 1 Statistics on lignocellulose content of *Miscanthus***

| Species | Lignocellulose | Cellulose | Hemicellulose | Lignin | Extracts | Total ash | H / L |
| --- | --- | --- | --- | --- | --- | --- | --- |
| *M. sinensis* | 77.94±6.06% | 37.66±3.80% | 22.94±3.71% | 17.35±1.29% | 15.83±5.02% | 2.47±0.57% | 3.51±0.36 |
| *M. ﬂoridulus* | 75.16±4.98% | 36.28±2.58% | 21.95±3.61% | 16.94±1.18% | 18.41±4.17% | 2.74±0.81% | 3.45±0.29 |
| *M. nudipes* | 75.68±3.02% | 36.07±1.51% | 22.39±2.70% | 17.21±0.55% | 21.10±1.77% | 2.51±0.81% | 3.40±0.24 |
| *M. sacchariﬂorus* | 83.71±4.78% | 39.25±3.06% | 26.35±3.73% | 18.11±1.35% | 11.62±3.81% | 2.51±0.48% | 3.64±0.34 |
| *M. lutarioriparius* | 81.50±5.23% | 39.96±3.96% | 22.85±3.95% | 18.69±1.49% | 12.43±3.84% | 2.43±0.65% | 3.37±0.22 |
| Hybrid | 74.72±7.13% | 37.14±3.98% | 21.21±3.53% | 16.37±1.95% | 20.67±6.43% | 2.56±0.72% | 3.60±0.46 |
| Average | 80.27±6.51% | 38.38±3.52% | 24.23±4.21% | 17.66±1.56% | 14.50±5.60% | 2.53±0.59% | 3.56±0.35 |
