# Supplementary material for: Natural Variation of Lignocellulosic Components in Miscanthus Biomass in China
Source: Front Chem. 2020 Nov 5;8:595143. doi: 10.3389/fchem.2020.595143 (PMC7674668; doi:10.3389/fchem.2020.595143)
Supplement: Supplementary file 2 [file Table_2.DOCX]

**Table 2 Coefficient of variation of lignocellulosic components of *Miscanthus***

| Species | *M. sinensis* | *M. ﬂoridulus* | *M. nudipes* | *M. sacchariﬂorus* | *M. lutarioriparius* | Hybrid |
| --- | --- | --- | --- | --- | --- | --- |
| Cellulose | 10.09% | 7.12% | 4.19% | 7.80% | 9.91% | 10.73% |
| Hemicellulose | 16.18% | 16.46% | 12.07% | 14.14% | 17.29% | 16.65% |
| Lignin | 7.44% | 6.98% | 3.22% | 7.45% | 7.98% | 11.91% |
| Extracts | 31.72% | 22.66% | 8.78% | 32.81% | 30.89% | 31.09% |
| Total ash | 23.14% | 29.36% | 32.33% | 19.04% | 26.88% | 28.09% |
| H/L | 10.36% | 8.45% | 7.02% | 9.29% | 6.48% | 12.87% |
