# Supplementary material for: Natural Variation of Lignocellulosic Components in Miscanthus Biomass in China
Source: Front Chem. 2020 Nov 5;8:595143. doi: 10.3389/fchem.2020.595143 (PMC7674668; doi:10.3389/fchem.2020.595143)
Supplement: Supplementary file 3 [file Table_3.DOCX]

**Table 3 Statistical results of lignocellulose fractions of different ploidies of *M. sacchariﬂorus* and *M. lutarioriparius***

| Component | Species | Ploid | Average value | Coefficient  of variation (%) | Minimum | Maximum |
| --- | --- | --- | --- | --- | --- | --- |
|  |  |  | (%) |  | (%) | (%) |
| Lignocellulose | *M. sacchariﬂorus* | 2 | 84.05±4.89 | 5.82 | 72.47 | 91.79 |
|  |  | 4 | 81.97±3.87 | 4.73 | 72.41 | 85.90 |
|  | *M. lutarioriparius* | 2 | 78.65±5.64 | 6.66 | 69.46 | 86.82 |
|  |  | 4 | 84.35±3.56* | 4.23 | 77.41 | 89.99 |
| Cellulose | *M. sacchariﬂorus* | 2 | 39.58±2.73 | 6.89 | 32.60 | 47.52 |
|  |  | 4 | 37.53±4.10 | 10.93 | 30.29 | 42.63 |
|  | *M. lutarioriparius* | 2 | 39.60±4.03 | 10.19 | 35.46 | 46.16 |
|  |  | 4 | 40.33±4.12 | 10.23 | 33.44 | 44.87 |
| Hemicellulose | *M. sacchariﬂorus* | 2 | 26.46±3.58 | 13.52 | 19.49 | 34.23 |
|  |  | 4 | 25.83±4.54 | 17.56 | 20.23 | 33.55 |
|  | *M. lutarioriparius* | 2 | 20.74±3.41 | 16.44 | 16.96 | 26.73 |
|  |  | 4 | 24.96±3.42* | 13.69 | 18.74 | 30.46 |
| Lignin | *M. sacchariﬂorus* | 2 | 18.01±1.40 | 7.78 | 14.75 | 23.75 |
|  |  | 4 | 18.6±0.92 | 4.97 | 17.14 | 20.28 |
|  | *M. lutarioriparius* | 2 | 18.31±1.87 | 10.21 | 16.06 | 21.71 |
|  |  | 4 | 19.07±0.97 | 5.10 | 17.78 | 20.31 |
| Holocellulos | *M. sacchariﬂorus* | 2 | 66.04±4.52 | 6.84 | 55.92 | 73.98 |
|  |  | 4 | 63.37±3.73* | 5.89 | 53.64 | 67.56 |
|  | *M. lutarioriparius* | 2 | 60.34±3.73 | 6.18 | 53.40 | 65.12 |
|  |  | 4 | 65.28±3.17* | 4.86 | 59.63 | 69.68 |
| Extracts | *M. sacchariﬂorus* | 2 | 11.42±4.03 | 35.25 | 5.38 | 23.51 |
|  |  | 4 | 12.64±2.27 | 17.99 | 9.28 | 17.35 |
|  | *M. lutarioriparius* | 2 | 14.29±4.05 | 28.33 | 8.10 | 19.41 |
|  |  | 4 | 10.57±2.71* | 25.62 | 7.23 | 16.46 |
| Total ash | *M. sacchariﬂorus* | 2 | 2.58±0.47 | 18.04 | 1.51 | 3.70 |
|  |  | 4 | 2.14±0.37** | 17.09 | 1.47 | 2.83 |
|  | *M. lutarioriparius* | 2 | 2.22±0.37 | 16.73 | 1.67 | 2.67 |
|  |  | 4 | 2.65±0.82 | 30.97 | 1.73 | 3.77 |
| H/L | *M. sacchariﬂorus* | 2 | 3.68±0.34 | 9.14 | 2.39 | 4.51 |
|  |  | 4 | 3.41±0.25* | 7.46 | 2.86 | 3.73 |
|  | *M. lutarioriparius* | 2 | 3.31±0.22 | 6.76 | 3.00 | 3.57 |
|  |  | 4 | 3.43±0.21 | 6.14 | 3.15 | 3.79 |

* Significant difference at P<0.05, ** Significant difference at P<0.01
