# Supplementary material for: Natural Variation of Lignocellulosic Components in Miscanthus Biomass in China
Source: Front Chem. 2020 Nov 5;8:595143. doi: 10.3389/fchem.2020.595143 (PMC7674668; doi:10.3389/fchem.2020.595143)
Supplement: Supplementary file 4 [file Table_4.DOCX]

**Table 4 Correlation analysis between lignocellulose content and geographical distribution of *M. sacchariﬂorus***

| *M. sacchariﬂorus* | latitude | | longitude | | altitude | |
| --- | --- | --- | --- | --- | --- | --- |
| N=85 | Pearson | Significance | Pearson | Significance | Pearson | Significance |
|  | correlation |  | correlation |  | correlation |  |
| Cellulose | 0.147 | 0.178 | -0.060 | 0.586 | 0.171 | 0.119 |
| Hemicellulose | 0.409^**^ | 0.000 | 0.242^**^ | 0.026 | -0.007 | 0.948 |
| Lignin | 0.162 | 0.138 | 0.113 | 0.304 | -0.080 | 0.466 |
| Holocellulose | 0.441^**^ | 0.000 | 0.160 | 0.145 | 0.111 | 0.311 |
| Extracts | -0.523^**^ | 0.000 | -0.346^**^ | 0.001 | 0.095 | 0.389 |
| Total ash | 0.361^**^ | 0.001 | 0.342^**^ | 0.001 | -0.094 | 0.393 |
| H/L | 0.239^*^ | 0.028 | 0.073 | 0.507 | 0.131 | 0.234 |
